# Rapamycin Maintains the Chondrocytic Phenotype and Interferes with Inflammatory Cytokine Induced Processes

**DOI:** 10.3390/ijms18071494

**Published:** 2017-07-11

**Authors:** Andrea De Luna-Preitschopf, Hannes Zwickl, Stefan Nehrer, Markus Hengstschläger, Mario Mikula

**Affiliations:** 1 Center for Regenerative Medicine and Orthopedics, Danube University Krems, 3500 Krems, Austria; stefan.nehrer@donau-uni.ac.at; 2 Department of Internal Medicine 2, University Hospital Krems, Karl Landsteiner University of Health Sciences, 3500 Krems, Austria; hannes.zwickl@kl.ac.at; 3 Center for Pathobiochemistry and Genetics, Medical University of Vienna, 1090 Vienna, Austria; markus.hengstschlaeger@meduniwien.ac.at (M.H.); mario.mikula@meduniwien.ac.at (M.M.)

**Keywords:** mTORC1, rapamycin, inflammatory cytokines, osteoarthritis

## Abstract

Osteoarthritis (OA) is hallmarked by a progressive degradation of articular cartilage. Besides risk factors including trauma, obesity or genetic predisposition, inflammation has a major impact on the development of this chronic disease. During the course of inflammation, cytokines such as tumor necrosis factor-alpha(TNF-α) and interleukin (IL)-1β are secreted by activated chondrocytes as well as synovial cells and stimulate the production of other inflammatory cytokines and matrix degrading enzymes. The mTORC1 inhibitor rapamycin is a clinical approved immunosuppressant and several studies also verified its chondroprotective effects in OA. However, the effect of blocking the mechanistic target of rapamycin complex (mTORC)1 on the inflammatory status within OA is not well studied. Therefore, we aimed to investigate if inhibition of mTORC1 by rapamycin can preserve and sustain chondrocytes in an inflammatory environment. Patient-derived chondrocytes were cultured in media supplemented with or without the mTORC1 inhibitor rapamycin. To establish an inflammatory environment, either TNF-α or IL-1β was added to the media (=OA-model). The chondroprotective and anti-inflammatory effects of rapamycin were evaluated using sulfated glycosaminoglycan (sGAG) release assay, Caspase 3/7 activity assay, lactate dehydrogenase (LDH) assay and quantitative real time polymerase chain reaction (PCR). Blocking mTORC1 by rapamycin reduced the release and therefore degradation of sGAGs, which are components of the extracellular matrix secreted by chondrocytes. Furthermore, blocking mTORC1 in OA chondrocytes resulted in an enhanced expression of the main chondrogenic markers. Rapamycin was able to protect chondrocytes from cell death in an OA-model shown by reduced Caspase 3/7 activity and diminished LDH release. Furthermore, inhibition of mTORC1 preserved the chondrogenic phenotype of OA chondrocytes, but also reduced inflammatory processes within the OA-model. This study highlights that blocking mTORC1 is a new and promising approach for treating OA. Low side effects make rapamycin an attractive implementation to existing therapeutic strategies. We showed that rapamycin’s chondroprotective property might be due to an interference with IL-1β triggered inflammatory processes.

## 1. Introduction

The chronic degenerative joint disease Osteoarthritis (OA) is the single most common cause of disability in older adults [1]. It is characterized by disruption of the articular cartilage, degradation of the extracellular matrix (ECM) secreted by chondrocytes, remodeling of the subchondral bone, reduced cellularity within cartilage and swelling of the joint [2,3]. Major factors that promote the onset of OA include obesity, genetic predisposition, trauma, muscle weakness, physical activity, bone density and nutritional status [4,5]. The influence of the immune system on the development and progression of OA is one major factor in the pathogenesis of this disease [2]. In healthy cartilage, a balance between anabolic and catabolic processes is maintained by chondrocytes, the only cell type within articular cartilage [6,7]. OA is characterized by a disturbance of these processes, mainly evoked by inflammatory cytokines resulting in progressive degeneration of articular cartilage. The two most prominent inflammatory cytokines involved in OA are tumor necrosis factor-α (TNF-α) and interleukin-1β (IL-1β). Within the joints, they promote catabolic and destructive processes [8]. They are synthesized by chondrocytes, osteoblasts, cells forming the synovial membrane, and infiltrated mononuclear cells [9,10,11,12]. Both cytokines can stimulate their own expression in an autocrine manner, but also induce production of other inflammatory cytokines such as IL-6, IL-15, IL-17 and IL-18 in activated chondrocytes and synovial cells [13,14,15,16]. Importantly, they can also stimulate the production of matrix degrading enzymes. Activated chondrocytes produce matrix metalloproteinases (MMPs) with MMP-1, MMP-3, MMP-13 and a disintegrin and metalloproteinase (ADAM) with thrombospondin-1 domains (ADAMTS)-4 being the most prominent. Activation of these enzymes results in a progressive degradation of collagen type II, gylcosaminoglycans and proteoglycans (aggrecans) resulting in the onset of OA [17,18,19]. Anti-inflammatory cytokines (IL-4, IL-10 and IL-13) act as a counterpart to inflammatory cytokines. Their main function is to inhibit the synthesis of inflammatory cytokines, to increase proteoglycan synthesis, to block apoptosis of chondrocytes and to inhibit synthesis and secretion of MMPs [8]. They are also present in the pathophysiology of OA, but the impact of inflammatory cytokines is more dominant than the one of anti-inflammatory ones.

The mechanistic target of rapamycin (mTOR) is activated by growth factors, oxygen and nutrients and the mechanistic target of rapamycin complex 1 (mTORC1) pathway is a major regulator of cellular processes such as proliferation, cell growth and survival [20,21,22]. The mTORC1 specific inhibitor rapamycin is a Food and Drug Administration (FDA) approved drug that was developed to inhibit T-cell proliferation and to preserve renal allografts [23]. In vivo and in vitro studies have shown that rapamycin has a beneficial effect on the treatment of OA. This was mainly due to the induction of autophagy, a protective mechanism that prevents articular cartilage from being degraded during OA [24,25,26,27]. Less is known about the immunomodulatory properties of mTORC1 inhibition in OA. Therefore, we established an OA-model, in which pellets of patient-derived OA chondrocytes were treated with either TNF-α or IL-1β to mimic an inflammatory environment comparable to the one present in OA. To assess the impact of mTORC1 blockade, chondrocyte pellets were additionally treated with rapamycin. We aimed to investigate whether blocking mTORC1 by rapamycin influences maintenance of the chondrogenic phenotype of OA chondrocytes including ECM production and chondrogenic marker expression. In addition, we studied whether blocking mTORC1 is able to reduce the impacts of the inflammatory cytokines TNF-α and IL-1β including induction of cell death and stimulation of production of MMPs and other inflammatory cytokines. As a last step, we sought to determine if rapamycin can preserve the chondrogenic phenotype in chondrocytes cultured in the OA-model.

## 2. Results

### 2.1. Blocking the Mechanistic Target of Rapamycin Complex (mTORC)1 Prevents Degradation of the Extracellular Matrix

**Figure 1:**
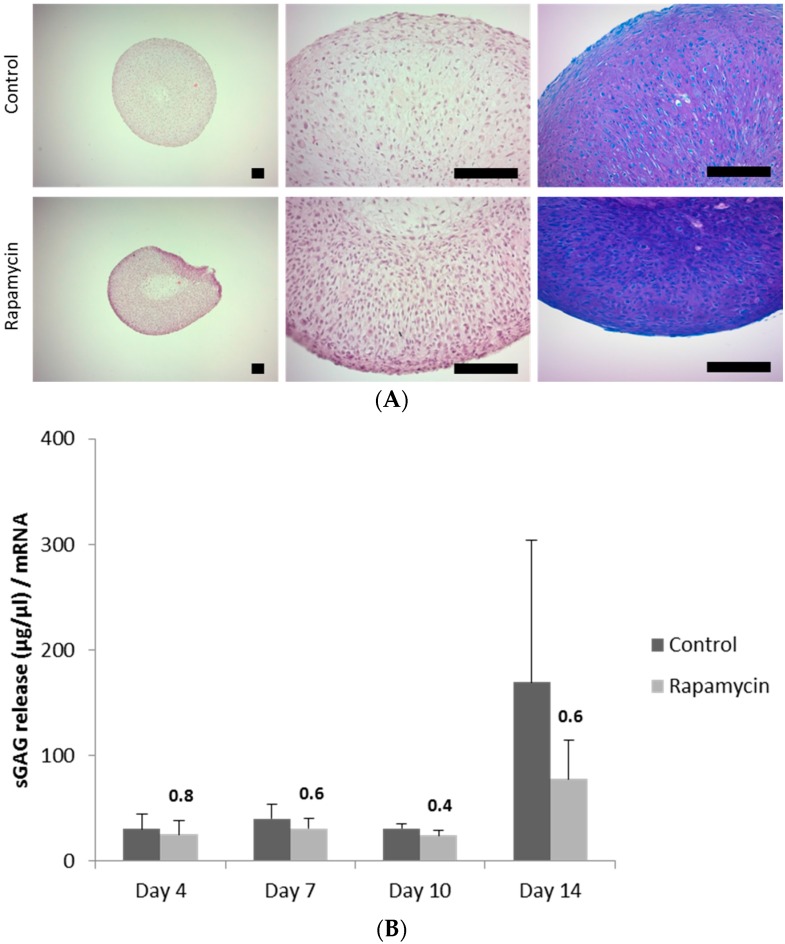
Blocking the mechanistic target of rapamycin complex (mTORC)1 prevents degradation of the extracellular matrix. (**A**) Patient-derived chondrocyte pellets were cultured in media supplemented without (control) or with the mTORC1 inhibitor rapamacin. Hematoxylin and eosin staining was performed to visualize the morphology of the patient-derived osteoarthritic (OA) chondrocyte pellets. Dimethylmethylene blue (DMMB) staining was performed indicating expression of sGAGs (purple staining). Scale bars = 80 µm; (**B**) sGAG release into supernatant of cultured pellets was measured on respective time points indicating degradation of extracellular matrix. Values are normalized to mRNA of pellets isolated on the respective time points. Exact values were given when *p* > 0.05.

**Figure A1:**
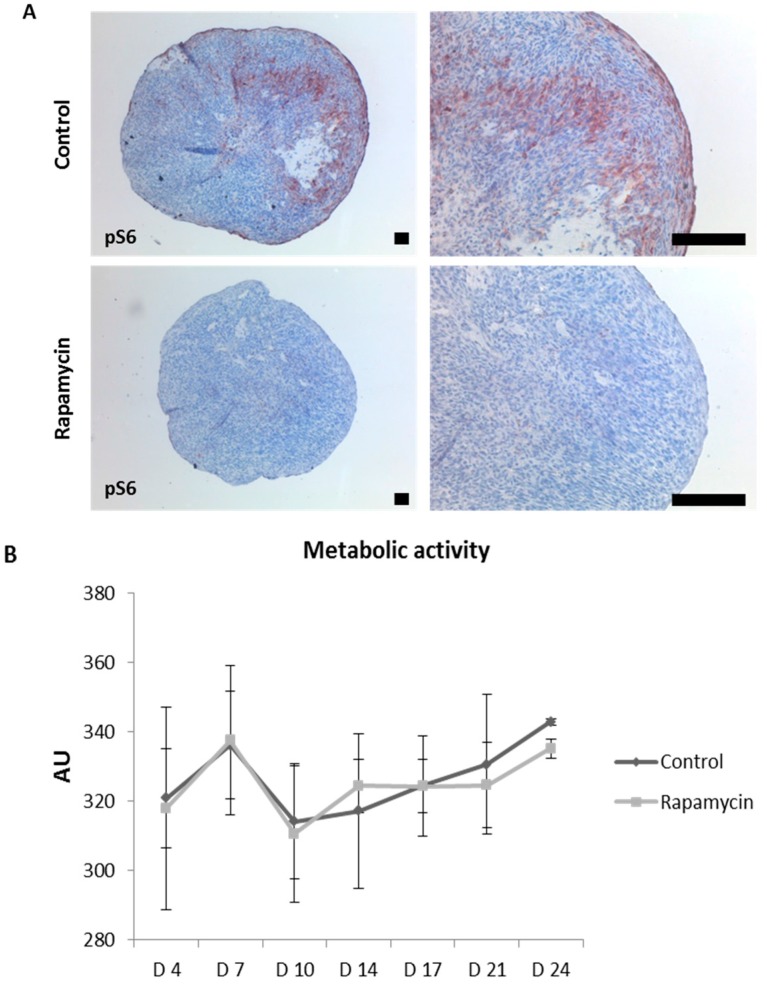
Effect of mTORC1 inhibition on metabolic activity of patient-derived OA chondrocytes. To verify effectiveness of rapamycin, patient-derived chondrocyte pellets were stained for the mTORC1 specific substrate pS6 (**A**) Alamar Blue assay was performed to measure impact of mTORC1 inhibition on the metabolic activity of patient-derived OA chondrocytes. Scale bars = 80µm; (**B**) Experiments were performed in triplicates and results are shown as arbitrary unit (AU) of respective ± SD.

Patient-derived OA chondrocytes were formed into three-dimensional aggregates (=pellets) (see Section 4) and were cultured in chondrogenic culture media supplemented with or without (control) the mTORC1 inhibitor rapamycin for 14 days. Staining of the mTORC1 specific substrate phospho-S6 (pS6) was performed to verify downregulation of mTORC1 upon rapamycin treatment (Figure A1A). As expected, hematoxylin and eosin staining demonstrated that cells are smaller upon rapamycin treatment as protein syntheses is affected upon this treatment. In addition, dimethylmethylene blue (DMMB) staining of pellets revealed that rapamycin enhances sGAG synthesis indicated by a darker purple staining compared to control pellets (Figure 1A). Blocking mTORC1 did not affect viability of chondrocytes within the pellets indicated by measurement of the metabolic activity (Figure A1B). Interestingly, upon rapamycin treatment, metabolic activity reached a plateau on Day 14 suggesting to stop our experiments on this time point. To investigate beneficial effects of rapamycin on cartilage maintenance, levels of secreted sGAGs into the supernatant were determined giving insights to what extend the extracellular matrix is degraded and subsequently released into the media (Figure 1B). Supernatants of pellets were collected on indicated time points and we observed that sGAGs were released in lower levels when mTORC1 was blocked. This effect was especially demonstrated on Day 14, as sGAG release levels were almost twice as high in control treated pellets compared to rapamycin treated ones, indicating that blocking mTORC1 can protect ECM from being degraded and therefore support cartilage maintenance.

### 2.2. Effects of mTORC1 Inhibition on the Chondrogenic Phenotype of Patient-Derived Osteoarhritic (OA) Chondrocytes

**Figure 2:**
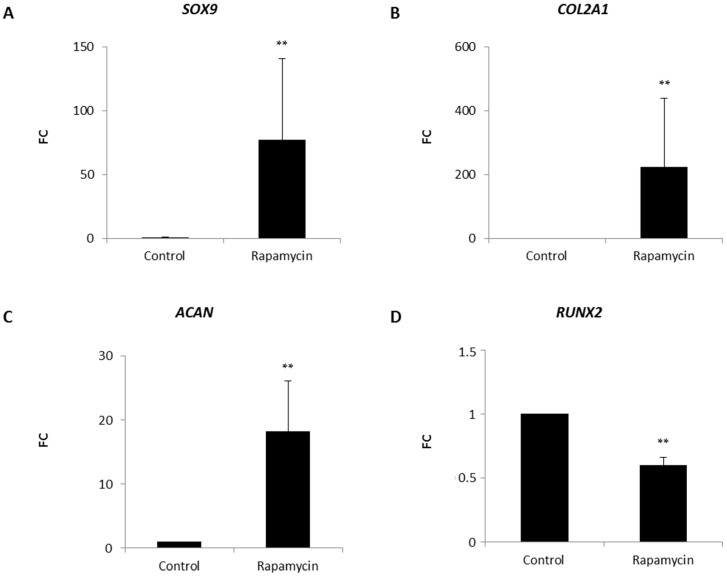
Effect of mTORC1 inhibition on the chondrogenic gene expression of patient-derived OA chondrocytes. Collagen type I-chondrocyte constructs were cultured in chondrogenic culture media supplemented without (control) with or rapamycin for 14 days. mRNA was isolated and quantitative real time polymerase chain reaction (RT-PCR) was performed: for the main chondrogenic markers *SRY-box* (*SOX*)*9*, *collagen type II α 1 chain* (*COL2A1*) and *aggrecan* (*ACAN*) (**A**–**C**); and for the hypertrophic marker *RUNX2*; (**D**) Experiments were performed in triplicates and results are shown as relative gene expression ± standard error of the mean (SEM) versus control. *p* < 0.01 was considered highly significant (**).

Patient-derived OA chondrocytes were casted into a collagen type I hydrogel. This approach is often used in matrix autologous chondrocyte implantation (MACI), one of the most common methods used to repair cartilage defects. Media was supplemented with or without (control) the mTORC1 inhibitor rapamycin. After 14 days of cultivation, cells were harvested and mRNA was isolated. Quantitative real time polymerase chain reaction (RT-PCR) demonstrated that blocking mTORC1 had a massive effect on the expression of the main chondrogenic markers. This inhibition resulted in a strong upregulation of the transcription factor *SRY-box* (*SOX)9* as well as of its target genes *COL2A1* and the proteoglycan *ACAN* (Figure 2A–C). Furthermore, we investigated the expression levels of *RUNX2*, a marker associated with the onset of hypertrophy of chondrocytes (Figure 2D) [28,29]. Interestingly, rapamycin was able to reduce levels of *RUNX2* compared to control treated pellets, indicating that mTORC1 inhibition has a beneficial effect on the maintenance of the chondrogenic phenotype and can be applied to MACI to further improve the quality of the newly formed cartilage.

### 2.3. mTORC1 Inhibition Prevents Chondrocytes from Undergoing Apoptosis

**Figure 3:**
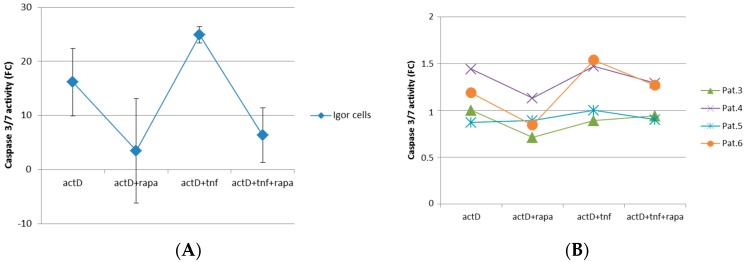
Caspase 3/7 assay of chondrocytes cultured in a monolayer under inflammatory conditions. To induce apoptosis, chondrocytes obtained from: healthy cartilage (**A**); and patient-derived OA chondrocytes (**B**) were cultured with the transcription inhibitor Actinomycin D. To mimic the inflammatory environment present in OA, chondrocytes were treated with tumor necrosis factor α (TNF-α). To assess protective effect of mTORC1 inhibition on OA chondrocytes, cells were also treated with rapamycin. Experiments were performed in triplicates and results are shown as mean ± standard deviation (SD) was calculated for (**A**).

To create an environment that is comparable to osteoarthritic conditions, chondrocytes were cultured as a monolayer in chondrogenic culture media containing the inflammatory cytokine TNF-α. To assess the role of mTORC1 regarding its protective role also in healthy cartilage, IGOR cells were obtained and implemented in this experiment (Figure 3A). As TNF-α alone could not induce apoptosis in chondrocytes, cells were also treated with the transcription inhibitor Actinomycin D. Effectiveness of Actinomycin D was demonstrated as Caspase 3/7 activity levels were strongly induced in chondrocytes (Figure 3A,B). Incubation of Actinomycin D together with rapamycin resulted in reduced Caspase 3/7 levels. As we expected, in the OA-model, treatment of Actinomycin D together with TNF-α led to a boost of Caspase 3/7 activity. Surprisingly, blocking mTORC1 was potent to decrease this induction indicating a rescuing effect of the mTORC1 inhibition on chondrocytes in the OA-model.

### 2.4. Block of mTORC1 Prohibits Cellular Cytotoxicity and Cytolysis of Patient-Derived OA Chondrocytes

**Figure 4:**
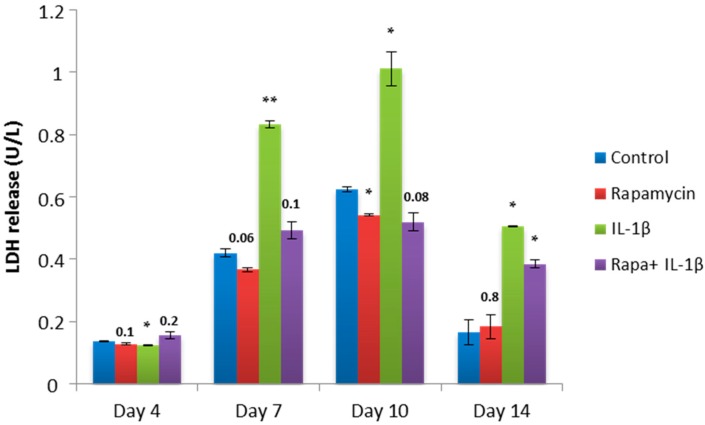
Lactate dehydrogenase (LDH) release of chondrocyte pellets cultured in an OA-model. Patient-derived OA chondrocyte pellets were cultured in media supplemented with or without rapamycin, the inflammatory cytokine interleukin (IL)-1β or rapamycin in combination with IL-1β for 14 days. Chondrocyte pellets cultured with culture media alone are indicated as control. Supernatants were collected on indicated time points and released LDH was measured. Experiments were performed in triplicates and results are shown as mean ± SEM versus control. *p* < 0.05 was considered significant (*) and *p* < 0.01 was considered highly significant (**). Exact values were given when *p* > 0.05.

To further confirm the rescuing property of mTORC1 inhibition, we performed a lactate dehydrogenase (LDH) assay that measures LDH released into the media from damaged cells as a biomarker for cellular cytotoxicity and cytolysis. This time, pellets were treated with the inflammatory cytokine IL-1β. Additionally, pellets were also cultivated with rapamycin to assess the impact of mTORC1 inhibition on the inflammatory state. On Days 4, 7, 10 and 14, supernatants were collected and released LDH was measured (Figure 4). In the control pellets, blocking mTORC1 reduced LDH levels compared to untreated pellets. High levels of LDH were measured in those pellets which were treated with IL-1β with a peak on Day 10. Surprisingly, combination of IL-1β and rapamycin resulted in a drop of LDH release to levels comparable to the group which was treated with rapamycin alone. This finding suggests that mTORC1 inhibition has a protective effect on chondrocytes within an OA-model and that it can re-establish the conditions found in control pellets.

### 2.5. Blocking mTORC1 Promotes Chondrogenesis and Suppresses Cartilage Degrading and Inflammatory Processes in an OA-Model

**Figure 5:**
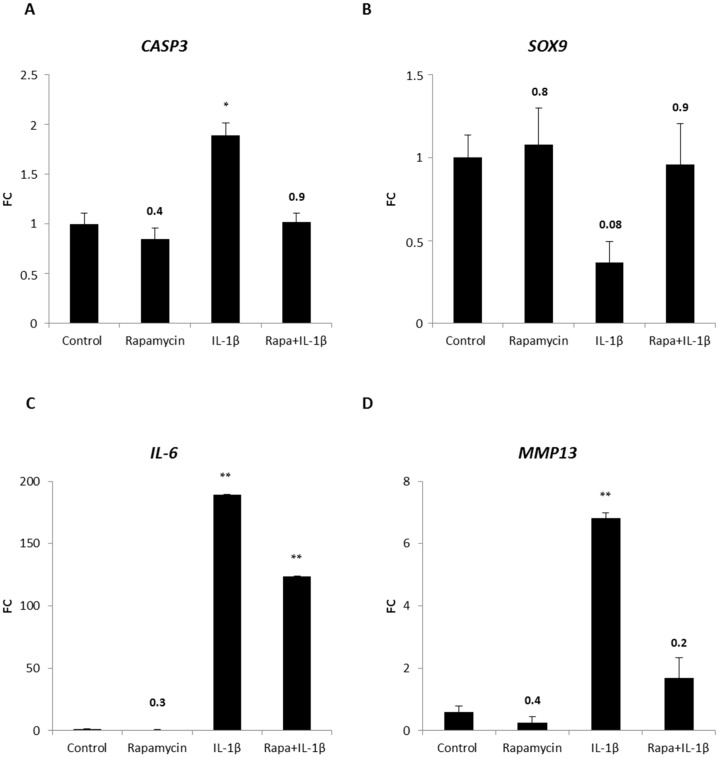
Chondroprotective and anti-inflammatory effect of mTORC1 inhibition on patient-derived OA chondrocyte pellets in an OA-model. Chondrocyte pellets were cultured with media containing rapamycin, IL-1β or rapamycin in combination with IL-1β for 14 days. Chondrocyte pellets cultured in chondrogenic culture media alone are indicated as control. *CASP3*, *SOX9*, *IL-6* and *MMP13* mRNAs were analyzed by quantitative real time polymerase chain reaction (RT-PCR) (**A**–**D**). Experiments were performed in triplicates and results are shown as fold change (FC) of respective gene expression ± SEM versus control. *p* < 0.05 was considered significant (*) and *p* < 0.01 was considered highly significant (**). Exact values were given when *p* > 0.05.

To verify this finding, levels of the apoptotic marker cleaved caspase-3 were investigated in chondrocytes within the OA-model (Figure 5A). *Cyseinyl-aspartate specific proteinase* (*CASP*)*3* levels of rapamycin treated pellets were comparable to those present in untreated chondrocyte pellets. As observed in the LDH assay, IL-1β induced cell death, demonstrated by an increase of *CASP3* expression. Again, when rapamycin was added to the OA-model, the levels of the apoptotic marker were reversed confirming the assumption of the rescuing property of mTORC1 blockade. Since rapamycin showed beneficial effects on the expression of chondrogenic genes in a cell-collagen type I construct (Figure 2A–C), we sought to investigate the impact of mTORC1 inhibition on the chondrogenic state within the OA-model. Therefore quantitative RT-PCR of the main chondrogenic transcription factor *SOX9* was performed (Figure 5B). Blocking mTORC1 resulted in a slight increase of *SOX9* levels compared to control groups, but presence of the inflammatory cytokine IL-1β led to a strong reduction of *SOX9* expression. Interestingly, IL-1β together with rapamycin induced again *SOX9* expression comparable to untreated pellets. This result highlighted the chondroprotective property of mTORC1 blockade on chondrocytes within the OA-model. To verify the effectiveness of our OA-model, we investigated if IL-1β induces other inflammatory cytokines as it does in OA [8]. Hence, we determined the amount of *IL-6*, a target of IL-1β, in our system (Figure 5C). Low levels of *IL*-6 were detected in untreated pellets and pellets cultured with rapamycin. As expected, in our OA-model, IL-1β strongly induced *IL-6* expression. Interestingly, rapamycin could reduce this induction verifying the anti-inflammatory property of rapamycin in our OA-model. Next, we determined if rapamycin can affect enzymes that are known to be induced by inflammatory cytokines and that mediate degradation of cartilage during the course of OA. Therefore, we evaluated the levels of *MMP13*, an enzyme that degrades type II collagen (Figure 5D). Rapamycin was able to reduce the amount of *MMP13* to levels lower than detected in control pellets. As MMPs are activated in an inflammatory state, presence of IL-1β leads, as we expected, to a massive induction of *MMP13* expression. Surprisingly, addition of rapamycin to the IL-1β treated pellets resulted in a strong decline of *MMP13* levels demonstrating that rapamycin is able to protect ECM from being degraded.

## 3. Discussion

In this study, we aimed to investigate the chondroprotective and anti-inflammatory effects of the inhibition of mTORC1 by rapamycin on OA chondrocytes. Within the cartilage, chondrocytes secrete an ECM which is mainly composed of type II collagen and proteoglycans [30]. Onset of OA is hallmarked by a degradation of the ECM by MMPs and ADAMTS and, as a result, components of the respective ECM proteins are released into the synovial fluid that surrounds the joints. Proteoglycans such as aggrecans consist of a core protein that is covalently bound to various GAGs. Upon proteoglycan degradation GAGs are released and their amount is proportional to the degree of degradation. We could show that rapamycin was able to reduce sGAG release levels compared to control group. The mTORC1 pathway is a major regulator of crucial cellular processes including proliferation, survival and cell growth. In human OA cartilage, mTORC1 is overexpressed and in vitro studies in which OA was surgically induced showed that blocking mTORC1 by rapamycin protects cartilage from being degraded [31]. One explanation is that rapamycin stimulates autophagy, which improves the survival of human articular chondrocytes [24,25,31,32]. Furthermore, the beneficial outcome of mTORC1 inhibition on the chondrogenic differentiation of human stem cells has been reported [33].

We established an OA-model, in which patient-derived OA chondrocytes were formed into three dimensional pellets and cultured in media containing either TNF-α or IL-1β. As neutrophiles are absent in the synovial fluid, OA has been stated to be a non-inflammatory arthropathy [34]. However, in the last decade, in vitro and in vivo studies have demonstrated more and more the impact of inflammation on the pathophysiology of OA. TNF-α and IL-1β have been considered as the main inflammatory cytokines triggering cartilage destruction, the hallmark of OA [2]. Due to abnormal mechanical and oxidative stress, these two cytokines are produced by all cells of the joint, including activated chondrocytes and synovial fibroblasts [7,35]. We demonstrated that rapamycin had chondroprotective effects within the OA-model indicated by reduced LDH and Caspase 3/7 levels as well as by a reduced expression of the matrix degrading enzyme *MMP13* and the inflammatory cytokine *IL-6*. It is known that inflammatory cytokines can induce chondrocyte death by reducing the efficiency of the respiratory chain leading to enhanced mitochondrial dysfunction in OA [8]. Furthermore, expression of endoplasmatic reticulum (ER) stress-associated molecules is induced by TNF-α and IL-1β resulting in chondrocyte death [36]. Rapamycin might have a protective effect preventing damage of these cellular organelles. All these findings suggest that blocking mTORC1 by rapalogs such as rapamycin or everolimus might be a new approach to reduce inflammatory-induced progression of OA. Rapamycin seems to have similar effectiveness as anti-inflammatory cytokines such as IL-4, IL-10 and IL-13. Just as these cytokines, rapamycin inhibits the synthesis of inflammatory cytokines as well as increases proteoglycan synthesis, inhibits apoptosis of chondrocytes and decreases synthesis and secretion of metalloproteinases. Although this study possesses some limitations as this is only an in vitro study, effects of TNFα, IL-1β as well as of rapamycin on patient-derived OA chondrocytes can be evaluated within this system. All this gained knowledge must be implemented into existing therapies to develop new therapeutic strategies for OA in which mTORC1 inhibitors could be a promising pharmacological agent.

Currently treatment options for OA focus on the reduction of inflammation and pain of the patients and, depending on the degree of the disease, non-pharmacological, pharmacological and surgical interventions are applied [37,38,39]. Moderate exercises, regular physical activity and weight loss reduce signs of OA, decrease systemic inflammation and lead to strengthening of the muscle which acts as a supporter around the joints. Additionally, the use of orthotics helps to reduce the pain [35,40,41]. For pharmacological interventions, non-steroidal anti-inflammatory drugs (NSAIDs) in combination with gels or transdermal patches containing lidocaine or paracetamol result in reduced pain caused by inflammation. Despite the beneficial outcome of these drugs, gastrointestinal, renal, and cardiovascular side-effects are the main challenges that have to be overcome [42,43,44]. If neither non-pharmacological nor pharmacological interventions lead to the desired outcome, surgical procedures including joint replacement surgery or osteotomy have to be conducted [35]. Rapamycin is a promising and potent therapeutic agent and its application is accompanied with low donor site morbidity. Combination with other pharmacological drugs including interleukin-1 receptor antagonist (IL-1Ra), aggrecanase inhibitors or anti-IL-1β and anti-TNF-α antibodies have to be extensively investigated. In addition, signaling pathways activated by inflammatory cytokines including nuclear factor κ-light-chain-enhancer of activated B-cells (NF-κB) or mitogen-activated protein kinase (MAPK) signaling have to be better studied with regard to establish new therapy approaches which will palliate the suffering and bring back quality of life of affected patients.

## 4. Materials and Methods

### 4.1. Isolation of Patient-Derived Osteoarthritic (OA) Chondrocytes

Human articular cartilage was obtained from four osteoarthritic patients from the Universitätsklinikum Krems. An informed consent of the patients was obtained and the institutional review board of the Danube University Krems (GS4-EK-4/199-2013) approved on 2 July 2013 the study. For the isolation of chondrocytes, the macroscopic damaged cartilage was removed from the femoral condyles. Cartilage samples were cut into 2–3 mm^3^ pieces followed by enzymatic digestion with 0.2 WU/mL Liberase (Roche Diagnostics GmbH, Mannheim, Germany) diluted in media (GIBCO DMEM /F12 GlutaMAX TM-I, Invitrogen, LifeTech Austria, Vienna, Austria) supplemented with antibiotics (100 U/mL penicillin; 0.1 mg/mL streptomycin and 2.5 µg/mL amphotericin B (Sigma-Aldrich Chemie GmbH, Steinheim, Germany)). After 22 h at 37 °C incubation, chondrocyte suspension was strained through a 40 µm Cell Strainer (BD Biosciences, Franklin Lakes, NJ, USA) for removal of undigested cell debris. Next, cells were washed with phosphate-buffered saline (PBS) (PAA Laboratories GmbH, Linz, Austria) and centrifuged for 5 min with 500× *g* at room temperature. Afterwards, cells were resuspended in culture media (see above) containing 10% fetal calf serum (FCS) (PAA Laboratories GmbH, Linz, Austria) supplemented with 50 µg/mL ascorbic acid (Sigma-Aldrich). Chondrocytes were cultured in 75 cm^2^ cell culture flasks (Nunc, Rochester, NY, USA) at a density of 1 × 10^4^ cells/cm^2^ at 37 °C in 5% CO_2_ and media was changed every three days.

### 4.2. Cultivation of Patient-Derived OA Chondrocytes within a Collagen Type I Hydrogel

10 mg collagen type I solution (BD Biosciences) was diluted to 2.5 mg/mL in a neutral buffer containing 10× PBS in ultra-pure distilled water, and 1 N NaOH was used to adjust pH to 7.4. Patient-derived OA chondrocytes were expanded, trypsinized and 4 × 10^4^ cells were encapsulated in the collagen type I solution. The Cell-collagen type I solution was cast in a 1 mL volume/construct onto 24 well cell culture plates and was polymerized at 37 °C in 5% CO_2_ for 30–60 min. Post-polymerization, constructs were cultivated in chondrogenic culture media containing DMEM (Invitrogen) supplemented with 0.5% fetal calf serum, 1% insulin-transferrin-selenium (ITS) (i.e., recombinant human insulin, human transferrin, and sodium selenite; Sigma-Aldrich Chemie GmbH) 100 nm dexamethasone (Sigma-Aldrich Chemie GmbH), 50 µg/mL ascorbic acid (Sigma-Aldrich Chemie GmbH), 100× nonessential amino acids (GE Healthcare, Chicago, IL, USA), 1mM sodium pyruvate (Sigma-Aldrich Chemie GmbH), 5 ng/mL transforming growth factor β-1 (TGFβ-1) (Peprotech, Rocky Hill, NJ, USA), and 4% methyl cellulose (Sigma-Aldrich Chemie GmbH). To assess the effects of mTORC1 inhibition, media was supplemented with 25 nm rapamycin (Merck Millipore, Billerica, MA, USA). Constructs were cultured for 14 days and media was changed every 3 days. Rapamycin was added to the media upon preparation when chondrogenic culture media was changed. For isolation of chondrocytes from the collagen type I hydrogel, constructs were digested overnight using 25 U/mL proteinase K. Cell suspension contained living cells from which mRNA was isolated for further analyses.

### 4.3. Cultivation of Patient-Derived OA Chondrocytes in a Three Dimensional Aggregate

Chondrocytes were cultured until 80% confluent. Cells were harvested using 0.25% trypsin/Ethylenediaminetetraacetic acid (EDTA) (GE Healthcare) and cell number was assessed using cell counter and analyser system (CASY) measurement (Roche Diagnostics, Risch-Rotkreuz, Switzerland). In total, 2.5 × 10^5^ cells were resuspended in chondrogenic culture media. Next, cells were centrifuged with 1.500× *g* for 10 min at room temperature and after 24–48 h cells assembled into three-dimensional aggregates (=pellets). Pellets were cultured for 14 days at 37 °C in 5% CO_2_ and the media was changed every three days. To mimic the inflammatory state present in OA, 10 ng/mL IL-1β (Peprotech) or 10 ng/mL TNF-α (Peprotech) was added freshly to the media every three days (=OA-model). To assess the effect of mTORC1 inhibition on OA chondrocytes, 25 nm rapamycin was added to the media upon preparation and was freshly added when chondrogenic culture media was changed.

### 4.4. IGOR Cells

IGOR cells, which were isolated from healthy cartilage, were cultured in DMEM/F12 GlutaMAX TM-I media (Invitrogen) supplemented with antibiotics (100 U/mL penicillin; 0.1 mg/mL streptomycin and 2.5 µg/mL amphotericin B (Sigma-Aldrich Chemie GmbH)). These cells were a kind gift of the Universitätsklinikum Krems.

### 4.5. Hematoxylin and Eosin Staining

Patient-derived OA chondrocyte pellets were fixed dehydrated and embedded in paraffin. Paraffin was removed using xylol, isopropanol, and an alcohol gradient in descending order (all from Carl Roth, Karlsruhe, Germany). Sections were stained for 2 min in Mayer’s Hematoxylin solution (Carl Roth) followed by a washing step in tap water. Subsequently, sections were incubated in 50% and 70% ethanol for 2 min respectively and counterstained in Eosin solution (Carl Roth) for 45 s. Afterwards sections were dehydrated using an ascending alcohol gradient followed by xylol incubation. Sections were mounted using entellan (Carl Roth).

### 4.6. Immunohistochemical Staining

Immunohistochemical staining of patient-derived OA chondrocytes was performed as previously described [33]. As primary antibody, pS6 (1:150; Cell Signaling Technology, Danvers, MA, USA) was used.

### 4.7. 1,9 Dimethylmethylen Blue (DMMB) Staining

DMMB staining of patient-derived OA chondrocyte pellets was performed as previously described [33].

### 4.8. Sulfated Glycosaminoglycan (sGAG) Assay

The Blyscan™ GAG assay (Biocolor, Belfast, Ireland) was performed according to the manufacturer’s protocol. Supernatant was tested for released sGAG on indicated time points giving information about degradation of proteoglycans. Released sGAG was normalized to mRNA of pellets isolated on the respective time points.

### 4.9. Lactate Dehydrogenase (LDH) Assay

The lactate dehydrogenase assay (Promega, Madison, WI, USA) was performed according to the manufacturer’s protocol. 

### 4.10. Caspase 3/7 Assay

Chondrocytes were plated as a monolayer in a 96-well plate at a density of 1.5 × 10^4^ cells/well and were treated with 10 ng/mL TNF-α. To induce apoptosis, cells were also treated with 200 ng/mL Actinomycin D (Sigma-Aldrich Chemie GmbH) for four hours. Cysteinyl-aspartate specific protease 3/7 (Caspase 3/7) activation was assessed using Caspase-Glo 3/7 assay (Promega) according to the manufacturer’s protocol.

### 4.11. Alamar Blue Assay

The Alamar blue assay (Promega) was performed according to the manufacturer’s protocol.

### 4.12. RNA Extraction and Polymerase Chain Reaction

After 14 days of cultivation, total RNA was extracted from the chondrocyte pellets and the cells isolated from the collagen type I constructs according to the manufacturer’s protocol (Peqlab, Erlangen, Germany). cDNA synthesis and polymerase chain reaction (PCR) was performed as described [45]. Β-actin was used for normalization and relative gene expression was assessed using the comparative *C*_t_ method (2^−ΔΔ*C*t^). Human primers were designed using Primer3 v. 0.4.0 (http://bioinfo.ut.ee/primer3-0.4.0/) and are listen in Table 1.

### 4.13. Statistical Analysis

Chondrocytes from four different patients were obtained. For sGAG assay, LDH assay and RT-PCR, data were averaged and are presented as mean ± standard error of the mean (SEM). Statistical significance was tested by Student’s unpaired *t* test, comparing each experimental setup with the control (=untreated cells). GraphPad Prism version 4.00 software (GraphPad Software, San Diego, CA, USA) was used. *p* < 0.05 was considered significant (*) and *p* < 0.01 was considered highly significant (**). Exact values were given when *p* > 0.05.

## 5. Conclusions

The present results show that rapamycin is a potent agent to stimulate chondrogenesis and protect chondrocytes during the inflammatory events of OA. We showed that rapamycin prevents ECM degradation and that it can enhance the expression of the most prominent chondrogenic markers SOX9, COL2A1 and ACAN in patient derived OA chondrocytes embedded into a Collagen type-I hydrogel. Within an inflammatory environment, as present in OA, rapamycin can prevent apoptosis of OA chondrocytes as well as prohibit cellular toxicity of these cells. In addition, rapamycin is able to decrease the levels of inflammatory cytokines as well as of matrix degrading enzymes which lead to the symptoms connected with OA. Our observations suggest that rapamycin is not only an effective drug for the treatment of diseases such as Peutz-Jeghers syndrome, Cowden syndrome, Bannayan-Riley-Ruvalcaba syndrome, Lhermitte-Duclos disease, Proteus syndrome, von Hippel-Lindau disease, Neurofibromatosis type 1, and Polycystic kidney disease [22] but should also be considered as a new therapeutic approach for the treatment of diseases affecting articular cartilage. Its anti-inflammatory properties and its chondroprotective effects make rapamycin a new ray of hope in treating OA and therefore give back quality of life to those who are affected by this progressive disease.

## Figures and Tables

**Table 1:** Table of primers.

Gene	F-Primer	R-Primer
*SOX9*	5′-AGCGCCCCCACTTTTGCT-3′	5′-TGGCCGGGAAAGGCGAG-3′
*COL2A1*	5′- GATAAGGATGTGTGGAAGCCGGAGC-3′	5′-TCCTTTCTGTCCCTTTGGTCCTGGT-3′
*ACAN*	5′-TACACGCTACACCCTCGACTTTGA-3′	5′-TACGTCCTCACACCAGGAAACTCA-3′
*RUNX2*	5′-GTTACTGTCATGGCGGGTAACGAT-3′	5′-TCAAGCTTCTGTCTGTGCCTTCTG-3′
*MMP13*	5′-CCAGAAGTGCGGGGTAGGGG-3′	5′-TGTGTCCCATTTGTGGTGTGGGA-3′
*CASP3*	5′-CTCCTAGCGGATGGGTGCTATTGT-3′	5′-AGACCGAGATGTCATTCCAGTGCTT–3′
*IL6*	5′-GGCTGCTCCTGGTGTTTGCCT-3′	5′-TGCCAGTGCCTCTTTGCTGCT-3′
*ACTB*	5′-CTATCCAGGCTGTGCTATCCCTGT-3′	5′-CCTTAATGTCACGCACGATTTCC-3′

## Abbreviations

ADAMTS	A disintegrin and metalloproteinase with thrombospondin-1 domains
Caspase	Cyseinyl-aspartate specific proteinase
CASY	Cell counter and analyser system
DMMB	1,9 Dimethylmethylene blue
ECM	Extracellular matrix
EDTA	Ethylenediaminetetraacetic acid
ER	Endoplasmatic reticulum
FDA	Food and Drug Administration
FCS	Fetal calf serum
sGAG	Sulfated glycosaminoglycan
IL	Interleukin
ITS	Insulin-transferrin-selenium
LDH	Lactate dehydrogenase
MAPK	mitogen-activated protein kinase
MMP	Matrix metalloproteinase
mTOR	Mechanistic target of rapamycin
mTORC	Mechanistic target of rapamycin complex
NF-κB	Nuclear factor κ-light-chain-enhancer of activated B-cells
NSAID	Nonsteroidal anti-inflammatory drugs
OA	Osteoarthritis
pS6	Phospho-S6
RT-PCR	Real time polymerase chain reaction
SOX	SRY-box
TNF	Tumor necrosis factor
TGF	Transforming growth factor
